# IL-18 in atherosclerosis: context-dependent biology, human evidence, and translational challenges

**DOI:** 10.3389/fcvm.2026.1910314

**Published:** 2026-08-27

**Authors:** Dongshuai Zhang, Shuai Wang, Yang Li, Huijie Zhang, Bing Wang

**Affiliations:** 1 The First Affiliated Hospital of Heilongjiang University of Chinese Medicine, Harbin, Heilongjiang, China; 2 Heilongjiang University of Chinese Medicine, Harbin, Heilongjiang, China

**Keywords:** anti-inflammatory therapy, atherosclerosis, biomarker, inflammasome, interleukin-18, Mendelian randomization, translational medicine

## Abstract

Atherosclerosis is a chronic inflammatory disease shaped by interactions among vascular cells, innate immunity, adaptive immunity, and metabolic stress. Interleukin-18 (IL-18), an IL-1 superfamily cytokine activated predominantly downstream of inflammasomes, links vascular injury to type 1 immune responses. This structured narrative review integrates mechanistic, plaque-tissue, epidemiological, genetic, and therapeutic evidence for IL-18 in atherosclerosis. Experimental studies generally support pro-atherogenic actions, but these effects vary by plaque stage, cellular context, and genetic background. Human studies consistently show enrichment of IL-18 in inflamed plaques, whereas circulating IL-18 has limited independent diagnostic or prognostic performance after multivariable adjustment in several large cohorts. Drug-target Mendelian randomization supports associations with selected cardiovascular outcomes, but does not substitute for randomized clinical evidence and remains vulnerable to pleiotropy, instrument strength, ancestry, and lifelong-exposure assumptions. Direct IL-18 inhibitors have entered clinical development in inflammatory diseases, yet cardiovascular outcome efficacy has not been established. We therefore position IL-18 as a biologically plausible but unvalidated cardiovascular target and outline a stage-gated translational agenda centered on free IL-18 assays, plaque-context biomarkers, pharmacology, patient selection, and long-term safety.

## Introduction

Atherosclerosis, once considered primarily a lipid-driven disorder, is now recognized as a chronic inflammatory disease involving complex interactions between innate and adaptive immune mechanisms within the arterial wall (1). The landmark Canakinumab Anti-Inflammatory Thrombosis Outcomes Study (CANTOS) established interleukin-1β (IL-1β) as a therapeutic target for cardiovascular prevention. Canakinumab is a fully human monoclonal antibody that selectively neutralizes IL-1β; its use reduced major adverse cardiovascular events independently of lipid lowering (2). Nevertheless, IL-1β blockade only partially attenuated residual inflammatory risk and increased fatal infection and sepsis, motivating evaluation of additional targets within the inflammasome–IL-1 cytokine network.

Interleukin-18 (IL-18), originally identified in 1989 as an interferon-γ (IFN-γ)-inducing factor, represents a compelling yet underexplored candidate in atherosclerosis pathogenesis (3). Like IL-1β, IL-18 is an NLRP3 [NOD (nucleotide oligomerization domain)-, LRR (leucine-rich repeat)-, and PYD (pyrin domain)-containing protein 3] inflammasome-dependent cytokine that shares activation mechanisms with IL-1β but exhibits distinct transcriptional regulation, cellular distribution, and immunological functions, potentially underlying distinct pathogenic roles and therapeutic implications in atherosclerosis (4, 5). Unlike IL-1β, which requires *de novo* transcription following inflammatory stimulation, IL-18 is constitutively expressed in multiple cell types, including vascular smooth muscle cells (VSMCs), endothelial cells, and macrophages, positioning it as a preformed alarmin capable of rapid immune amplification (6, 7).

This review critically evaluates IL-18 rather than treating it as an isolated inflammatory biomarker. Its novelty lies in integrating plaque-stage and cell-specific biology with contradictory experimental evidence, large human cohorts, drug-target genetics, direct and upstream therapeutic programs, and practical barriers to cardiovascular translation. Particular attention is given to how IL-18 differs from IL-1β and IL-6, how it interacts with IFN-*γ*, TNF, CXCL9/CXCL10 and GM-CSF networks, and why circulating measurements may not represent biologically active IL-18 within plaques.

## Scope, novelty, and literature search

A structured narrative search was conducted in PubMed/MEDLINE, Europe PMC, and ClinicalTrials.gov from database inception through 21 July 2026. Search concepts combined “interleukin-18” or “IL-18” with “atherosclerosis”, “plaque”, “cardiovascular”, “coronary”, “stroke”, “heart failure”, “inflammasome”, “Mendelian randomization”, “single-cell”, “spatial transcriptomics”, “IL-18BP”, and individual therapeutic agents. Reference lists of key articles were screened. English-language primary human studies, mechanistic animal or cellular studies, genetic analyses, registered interventional trials, and high-quality reviews were eligible when they directly informed atherosclerosis biology or cardiovascular translation. Duplicates, reports without sufficient methodological detail, and IL-18 studies unrelated to cardiovascular mechanisms were excluded. Because study designs, exposures, assays, and outcomes were highly heterogeneous, evidence was synthesized narratively rather than pooled. This is a structured narrative review, not a systematic review or meta-analysis.

## IL-18

### The biology of IL-18

IL-18 is an IL-1 superfamily cytokine synthesized as a 24 kDa inactive precursor (pro-IL-18) that lacks a classical signal peptide and resides in the cytoplasm of producer cells (8). Maturation into the 17.2 kDa active form requires proteolytic cleavage, principally by inflammasome-activated caspase-1, including the NLRP3 inflammasome pathway (9, 10). Non-canonical processing can also occur through caspase-8 in selected death-receptor settings, granzyme B during cytotoxic lymphocyte interactions, proteinase 3 in neutrophil-rich inflammation, and mast-cell chymase in tissues enriched for activated mast cells (11–14). These context-specific routes broaden IL-18 activation beyond the canonical NLRP3–caspase-1 axis without implying that each route contributes equally in human plaques.

Mature IL-18 release is mechanistically coupled to gasdermin D (GSDMD)-mediated pore formation. Following inflammasome activation, caspase-1 cleaves GSDMD, releasing N-terminal cleavage products that oligomerize to form plasma membrane pores, thereby permitting IL-18 and IL-1β secretion (15). Beyond mere pore formation, electrostatic repulsion between the cytoplasmic face of GSDMD pores and proforms of IL-18 and IL-1β facilitates selective release of mature cytokines, whereas membrane repair may concurrently preserve cell viability (16).

### Distinction from IL-1β

A distinctive feature of IL-18 is its constitutive expression in multiple cell types without prior inflammatory priming. In contrast to IL-1β, which is typically absent from resting cells and requires Nuclear Factor Kappa B (NF-*κ*B)-dependent transcriptional induction, pro-IL-18 is basally expressed in macrophages, dendritic cells, endothelial cells, VSMCs, and adrenocortical cells (17). This constitutive pattern is particularly pronounced at epithelial barriers of the skin, respiratory tract, and gastrointestinal tract, where IL-18 contributes to homeostatic maintenance (18).

IL-18 transcription can be further increased by lipopolysaccharide (LPS), interferons, and microbial stimuli (19). In contrast to many LPS-inducible cytokines that decline after repeated exposure, pro-IL-18 expression may remain elevated in selected models of repeated endotoxin stimulation, indicating distinct regulation rather than universal resistance to tolerance (20). This property may be relevant to chronic low-grade stimulation in atherosclerosis. Independently of IFN-γ, IL-18 can also increase granzyme B expression and cytotoxic activity in NK cells and memory CD8+ T cells (21).

### IL-18 signaling

Upon maturation and release, IL-18 signals through a heterodimeric receptor complex, the IL-18 receptor (IL-18R), comprising IL-18Rα and IL-18Rβ (IL-18RAP) (22). IL-18 initially binds IL-18Rα with low affinity, and subsequent recruitment of IL-18Rβ generates a high-affinity functional receptor (23). The intracellular Toll/IL-1 receptor (TIR) domains of both receptor subunits undergo homotypic oligomerization, thereby recruiting the adaptor protein myeloid differentiation factor 88 (MyD88) through TIR–TIR domain interactions (24). MyD88 oligomerization initiates a signaling cascade involving IL-1R-associated kinases 4 (IRAK4), IRAK1/2, and tumor necrosis factor receptor-associated factor 6 (TRAF6), ultimately activating the NF-*κ*B and mitogen-activated protein kinases (MAPK) pathways, with downstream effects including cytokine production, cell proliferation, and effector programs (25–28).

The cellular distribution of the IL-18 receptor complex constrains IL-18 responsiveness. IL-18R is enriched in immune cells, particularly activated lymphocytes, but it is also detectable in vascular cells under inflammatory conditions. Naive T cells express little IL-18R; antigen encounter and cytokine exposure induce IL-18Rα and IL-18RAP, enabling IL-18 responsiveness (29). NK cells and several innate lymphoid cell subsets express the receptor complex. NK cells are developmentally and functionally related to innate lymphoid cells but should not be described simply as “NK-like ILCs”; depending on the classification framework, conventional NK cells are considered a distinct lineage or grouped within the broader ILC family. IL-18 amplifies type 1 immunity, defined here as IFN-γ-dominant cellular defense mediated mainly by NK cells, ILC1, Th1 cells, and cytotoxic CD8+ T cells (30). A non-canonical pathway involving the Na–Cl cotransporter SLC12A3 has also been reported in endothelial cells and adipocytes (31).

The potent pro-inflammatory activity of IL-18 is tightly regulated by endogenous antagonists. The biological activity of IL-18 is antagonized by the IL-18 binding protein (IL-18BP), a secreted glycoprotein that binds mature IL-18 with high affinity and prevents its interaction with IL-18Rα (32). IL-18BP functions as an endogenous soluble decoy receptor for IL-18, produced constitutively by diverse cell types and potently induced by IFN-*γ*, thereby forming a negative feedback loop. In healthy individuals, more than 95% of circulating IL-18 is bound to IL-18BP and rendered biologically inactive (23).

## Preclinical evidence of IL-18 in atherosclerosis

Multiple animal studies have established a pro-atherogenic role of IL-18. Elevated serum IL-18 levels were observed in Apoe^−^/^−^ mice with unstable compared to stable plaques, suggesting that IL-18 contributes to early plaque inflammation and modulates plaque stability and severity (33). In Apoe^−^/^−^ mice, *in vivo* blockade of IL-18 activity via IL-18BP overexpression attenuated atherosclerotic lesion development (34). Similarly, 24-week-old IL-18 deficient Apoe^−^/^−^ mice displayed markedly reduced atherosclerotic lesion size (35). The pro-atherogenic effects of IL-18 are mediated, in part, by its contribution to T-helper-1(Th1)-driven immunity. In synergy with IL-12, IL-18 stimulates naive CD4^+^ T cells to differentiate into IFN-γ-producing Th1 effector cells (36). Whitman et al. (37) reported that intraperitoneal IL-18 administration to Apoe^−^/^−^ mice aggravated atherosclerosis in an IFN-γ–dependent manner. Mechanistically, IL-18 activated NF-κB-dependent inflammatory signaling through the IL-18R, thereby promoting atherosclerotic plaque progression and destabilization (38). Furthermore, IL-18 is a potent pro-atherogenic factor that expands atherosclerotic lesions and enhances IFN-γ production and T-lymphocyte infiltration, promoting plaque destabilization (39).

Collectively, conventional Apoe^−^/^−^ studies support a pro-atherogenic role for IL-18. However, recent evidence demonstrates that this conclusion is not universal. In a JAK2V617F-driven clonal hematopoiesis model, antibody-mediated IL-18 neutralization increased early lesion size and advanced necrotic-core formation despite increasing plaque collagen. Reduced IFN-*γ* signaling, a shift from pyroptosis toward apoptosis, and impaired macrophage efferocytosis were implicated (40). Thus, IL-18 inhibition may alter different components of plaque stability in opposing directions, and effects may depend on genotype, disease stage, cell-death programs, and inflammatory milieu. This contradictory model is hypothesis-generating but directly argues against assuming uniform benefit across atherosclerosis phenotypes.

## Clinical evidence of IL-18's proatherogenic role

Mallat and colleagues first demonstrated that IL-18 is predominantly localized to macrophage-rich areas of the lipid core and is markedly elevated in both stable and unstable human carotid atherosclerotic plaques compared with controls (41). Thereafter, an immunohistochemical study showed that IL-18 and IL-18R were prominently expressed within human atherosclerotic plaques; furthermore, IL-18 expression was restricted to macrophages, whereas endothelial cells, smooth muscle cells and macrophages all expressed IL-18R (7). IL-18 enrichment in inflammatory foci, neovascular regions, and the fibrous cap underscores its role in plaque vulnerability.

Beyond local plaque expression, circulating IL-18 levels have been implicated in acute cardiovascular events. Elevated serum IL-18 levels were detected in patients with acute myocardial infarction, correlating with the severity of myocardial damage as well as in those with congestive heart failure (42, 43).

Despite these positive associations, circulating IL-18 has not shown consistent independent diagnostic value. In 1,111 community participants, IL-18 correlated with traditional risk factors and inflammatory markers, but adjustment for those covariates eliminated an independent association with subclinical carotid atherosclerosis (44). Similarly, among 2,231 asymptomatic Dallas Heart Study participants, IL-18 tracked cardiometabolic risk but did not independently improve assessment of atherosclerotic burden (45). These findings do not demonstrate biological irrelevance; rather, they indicate that circulating total IL-18 may add little beyond established risk factors for detecting subclinical disease. Population structure, assay variation, metabolic confounding, and the inability of total IL-18 to capture free or plaque-localized cytokine activity may contribute (46).

Evidence for prognosis is also mixed. Smaller studies reported higher IL-18 in acute coronary syndrome and in patients who later experienced adverse events (47–50). However, in 16,636 participants from the PLATO trial, the unadjusted association between IL-18 and cardiovascular death, spontaneous myocardial infarction, or stroke was attenuated after adjustment for clinical variables and biomarkers (adjusted hazard ratio 1.01, 95% confidence interval 0.98–1.04 per 25% higher IL-18) (49). Elevated IL-18 has also been described in atrial fibrillation, pulmonary hypertension, stroke, diabetes, and insulin resistance (51–55), but these associations may reflect shared inflammatory and metabolic burden. Accordingly, IL-18 should currently be regarded as a context-dependent research biomarker rather than an independently validated clinical diagnostic or prognostic test.

Drug-target Mendelian randomization (MR) has provided genetic evidence relevant to IL-18, but the signal is narrower than a general claim of cardiovascular protection. Brennan and colleagues used five independent cis-acting variants near IL18 from the SCALLOP consortium (*n* = 21,758) as proxies for lower circulating IL-18 (56). One standard-deviation lower genetically predicted IL-18 was associated with cardioembolic stroke [odds ratio (OR) 0.85, 95% CI: 0.79–0.92], peripheral artery disease (OR: 0.91, 95% CI: 0.84–0.97), atrial fibrillation (OR: 0.94, 95% CI: 0.89–0.99), and heart failure (OR: 0.84, 95% CI: 0.77–0.92), but not with coronary artery disease or non-cardioembolic stroke. Associations with downstream CRP, TNF, IFN-γ, and CXCL10 support biological relevance of the instrument, while cardiac imaging associations were small (57). Key human studies spanning plaque tissue, community cohorts, acute coronary syndromes, and drug-target genetic evidence are summarized in Table 1.

**Table 1:** Major human studies evaluating IL-18 in atherosclerotic or cardiovascular phenotypes.

Study	Design/sample	Outcome	Main estimate	Key limitation	Reference
Mallat et al.	Human carotid plaque tissue study	Stable vs. symptomatic/unstable plaque	Higher plaque IL-18 mRNA in symptomatic plaques (*P* < 0.01)	Tissue series; cross-sectional; cannot establish causality	(41)
CUDAS	Community cross-sectional cohort; *n* = 1,111	Subclinical carotid atherosclerosis	No independent association after multivariable adjustment	Single time-point total IL-18; subclinical phenotype	(44)
Dallas Heart Study	Population-based cross-sectional cohort; *n* = 2,231	Metabolic syndrome and subclinical atherosclerosis	Association attenuated after adjustment for traditional risk factors	Residual confounding; total circulating IL-18	(45)
PLATO biomarker study	ACS cohort; *n* = 16,636	CV death, spontaneous MI, or stroke at 1 year	Adjusted HR 1.01 (95% CI 0.98–1.04) per 25% higher IL-18	Acute-phase sampling; biomarker not treatment target	(49)
Brennan et al.	Drug-target cis-MR; exposure GWAS *n* = 21,758; outcome datasets *n* = 3,301–1,320,016	Multiple cardiovascular outcomes	Cardioembolic stroke OR 0.85; PAD 0.91; AF 0.94; HF 0.84 per SD lower IL-18	MR assumptions; pleiotropy; lifelong exposure; no direct CAD association	(56)

AF, atrial fibrillation; ACS, acute coronary syndrome; CAD, coronary artery disease; CV, cardiovascular; HF, heart failure; HR, hazard ratio; MI, myocardial infarction; MR, Mendelian randomization; OR, odds ratio; PAD, peripheral artery disease; SD, standard deviation.

MR inference depends on relevance, independence, and exclusion-restriction assumptions (74). Restricting variants to the IL18 locus reduces but does not abolish horizontal pleiotropy or linkage disequilibrium with neighboring regulatory signals. Other concerns include weak-instrument bias for secondary outcomes, winner's curse in protein quantitative trait loci, sample overlap, ancestry-specific transportability, multiple testing, and the assumption that lifelong modest genetically lower IL-18 reproduces the timing, magnitude, tissue penetration, and reversibility of pharmacological inhibition. Colocalization and sensitivity analyses strengthen interpretation but cannot prove that IL-18 itself mediates every association. The MR findings therefore justify target validation and trial design; they do not establish clinical efficacy.

Safety signals from MR require equally cautious interpretation. Lower genetically predicted IL-18 was associated with higher lung-cancer odds, whereas no association with infection was detected (56). A null genetic association does not exclude infection, vaccine-response, or immune-surveillance risks during high-intensity or long-term drug exposure, particularly because IL-18 participates in NK- and CD8-mediated antitumor immunity (58). The totality of genetic evidence supports further investigation of IL-18 modulation, particularly for heart failure, atrial fibrillation, and cardioembolic stroke pathways, while leaving direct atherosclerotic benefit unresolved.

## Context-dependent and cell-specific mechanisms of IL-18 across plaque evolution

**Figure 1:**
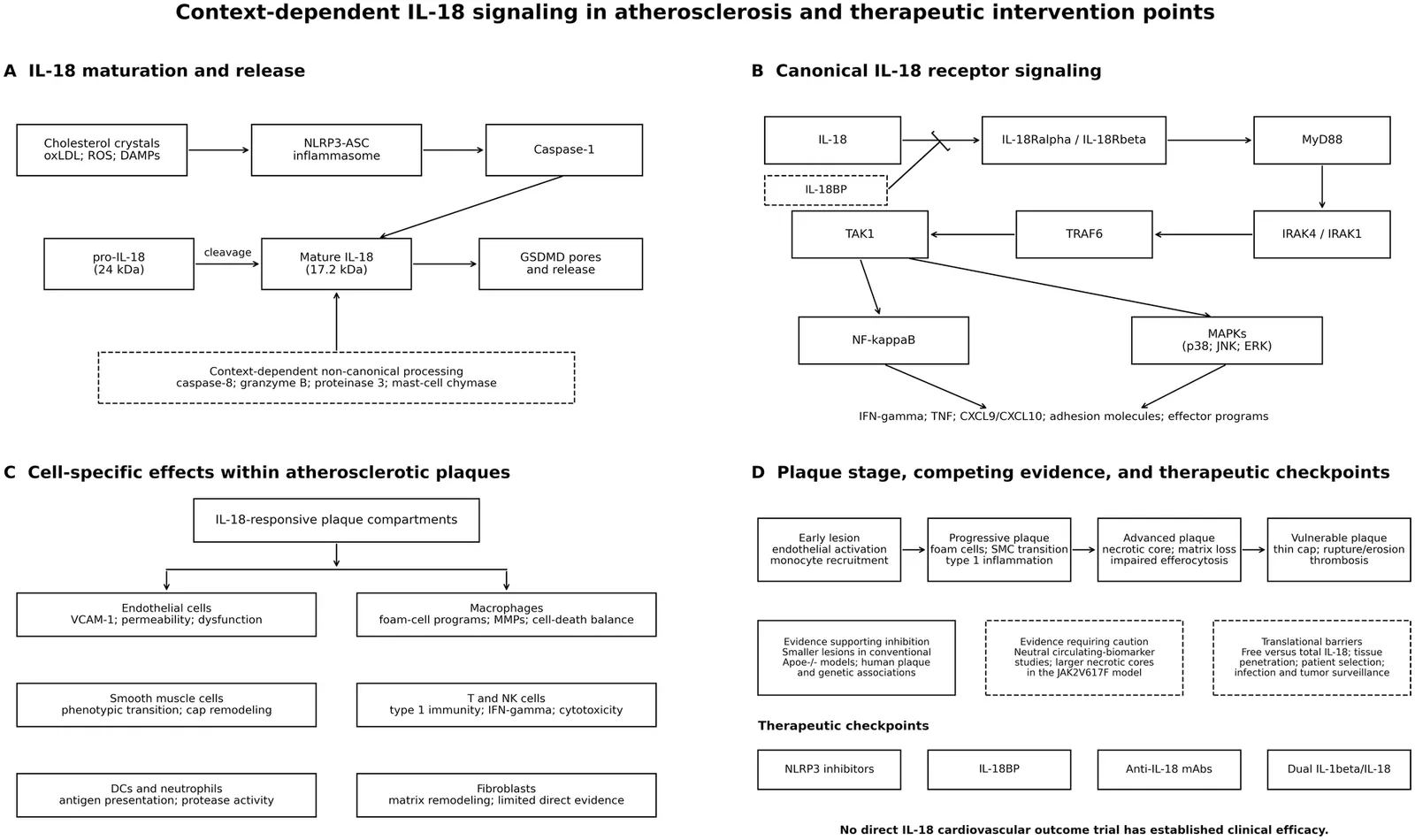
Context-dependent IL-18 signaling in atherosclerosis and therapeutic intervention points. **(A)** IL-18 maturation and release. Canonical NLRP3 inflammasome/caspase-1 signaling cleaves pro-IL-18 for GSDMD-dependent extracellular release. Context-dependent non-canonical IL-18 processing can involve caspase-8, granzyme B, and mast-cell chymase. **(B)** Canonical IL-18 receptor signaling. IL-18 engages IL-18Rα/IL-18Rβ to trigger the MyD88-IRAK-TRAF6-TAK1 cascade, activating downstream NF-κB and MAPK pathways. IL-18BP serves as an endogenous IL-18 inhibitor. **(C)** Cell-specific plaque responses. IL-18 regulates biological functions across multiple cell types within atherosclerotic plaques, including endothelial cells, macrophages, smooth muscle cells, T/NK cells, dendritic cells, neutrophils, and fibroblasts. **(D)** Plaque progression and therapeutic checkpoints. This panel depicts progressive atherosclerotic lesion development, conflicting preclinical evidence regarding IL-18 inhibition, and potential therapeutic strategies, including NLRP3 inhibitors, IL-18BP, anti-IL-18 monoclonal antibodies, and dual IL-1β/IL-18 blockade. No direct IL-18 cardiovascular outcome trial has established clinical efficacy. ASCVD, atherosclerotic cardiovascular disease; ASC, apoptosis-associated speck-like protein containing a CARD; CXCL9/10, C-X-C motif chemokine ligand 9/10; DAMPs, damage-associated molecular patterns; DCs, dendritic cells; ERK, extracellular signal-regulated kinase; GSDMD, gasdermin D; IFN-γ, interferon-gamma; IL-18BP, interleukin-18 binding protein; IRAK1/4, interleukin-1 receptor-associated kinase 1/4; JNK, c-Jun N-terminal kinase; MAPKs, mitogen-activated protein kinases; MMPs, matrix metalloproteinases; MyD88, myeloid differentiation primary response 88; NF-κB, nuclear factor kappa B; NK, natural killer; NLRP3, NLR family pyrin domain containing 3; oxLDL, oxidized low-density lipoprotein; ROS, reactive oxygen species; SMC, smooth muscle cell; TAK1, TGF-β-activated kinase 1; TNF, tumor necrosis factor; TRAF6, TNF receptor-associated factor 6; VCAM-1, vascular cell adhesion molecule-1.

Figure 1 summarizes IL-18 maturation, canonical IL-18R signaling, cell-specific responses, plaque-stage context, and therapeutic checkpoints. Cholesterol crystals, oxidized LDL (oxLDL), mitochondrial damage, and other danger signals activate inflammasomes, allowing caspase-1 to process pro-IL-18 and gasdermin D to facilitate release (60–64). Mature IL-18 binds IL-18Rα/IL-18Rβ, recruits MyD88, IRAK4/IRAK1, TRAF6 and TAK1, and activates NF-*κ*B and MAPK pathways (24–28). IL-18BP limits receptor engagement. In diabetic vascular inflammation, PKC-β-mediated reduction of IL-18BP may increase unopposed IL-18 activity and endothelial VCAM-1 expression (65).

The impact of this pathway changes across plaque evolution. In early lesions, endothelial activation and monocyte recruitment predominate; in progressive plaques, macrophage foam-cell programs, smooth-muscle-cell phenotypic transition, and chronic type 1 immunity become prominent; in advanced or vulnerable plaques, necrotic-core expansion, matrix degradation, failed efferocytosis, cap remodeling, rupture or erosion, and thrombosis become decisive. IL-18 can contribute to endothelial dysfunction, macrophage inflammatory programs, CD8+ T-cell and NK-cell IFN-γ production, and Th1 polarization (7, 32, 66, 67). Its effects on smooth muscle cells, dendritic cells, neutrophils, and fibroblast-like stromal cells are less uniformly defined and should not be inferred from bulk plaque measurements alone.

IL-18 may compromise plaque structural integrity by increasing extracellular-matrix turnover. In human monocytes it upregulates EMMPRIN and MMP-9, and vascular-cell studies implicate MMP-1, MMP-3, and MMP-9 in collagen degradation and fibrous-cap thinning (68, 69). IL-18-associated IFN-*γ* signaling can also modify regulatory T-cell stability and effector-cell balance (70). However, the JAK2V617F model indicates that greater collagen after IL-18 blockade can coexist with larger necrotic cores, underscoring that collagen content alone is an incomplete definition of plaque stability (40).

IL-18 further integrates inflammatory and metabolic pathways within the plaque microenvironment. The interplay between lipid metabolism and oxidative stress is integral to atherogenesis. Oxidative stress is a hallmark feature within atherosclerotic plaques, characterized by excessive reactive oxygen species (ROS) generation (71). Within plaques, ROS are not only direct mediators of tissue damage but also key triggers of innate immune signaling. Oxidized mitochondrial DNA (ox-mtDNA) is recognized by NLRP3 as a damage-associated molecular pattern (DAMP), thereby triggering assembly of the NLRP3-ASC-caspase-1 inflammasome complex and driving IL-18 maturation and release (72). IL-18 also promotes lipid oxidation and macrophage foam cell formation, exacerbating lipid accumulation and necrotic core expansion, thereby promoting plaque instability (73). IL-18 intersects with lipid metabolism through multiple pathways. In Apoe^−^/^−^ mice, IL-18 administration increases total cholesterol and LDL cholesterol while reducing HDL cholesterol, effects mediated via NF-κB-dependent upregulation of scavenger receptors and downregulation of liver X receptor α (LXR-α), a key regulator of cholesterol efflux (34). Accordingly, NF-κB inhibition with pyrrolidine dithiocarbamate (PDTC) blocks IL-18 signaling, attenuates inflammation, and restores plaque stability (38).

## Therapeutic targeting of IL-18: opportunities and challenges

Table 2 summarizes direct IL-18 neutralization, IL-18BP replacement, dual IL-1β/IL-18 blockade, and upstream inflammasome inhibition. Trial phase, mechanism, status, dosing route, indication, reported safety information, and cardiovascular relevance are distinguished. Most direct IL-18 programs have been developed for autoinflammatory or dermatologic disease, and no direct IL-18 cardiovascular outcome trial has established clinical efficacy.

**Table 2:** Clinical development of agents targeting IL-18 or upstream inflammasome signaling (registry status verified on 21 July 2026).

Agent	Target/mechanism	Phase and status	Indication	Route/regimen	Safety or limitation	Cardiovascular relevance	Trial ID
Tadekinig alfa	Recombinant human IL-18BP; sequesters free IL-18	Phase 2 completed	Adult-onset Still's disease	SC, 80 or 160 mg three times weekly	Injection burden; short inflammatory-disease exposure	No cardiovascular efficacy study	NCT02398435
Tadekinig alfa	Recombinant human IL-18BP	Phase 3 completed	NLRC4 mutation/XIAP deficiency	SC; registry protocol dosing	Very small monogenic-disease population	Pharmacology may not generalize to chronic ASCVD	NCT03113760
GSK1070806	Human anti-IL-18 IgG1 monoclonal antibody	Phase 1 completed	Atopic dermatitis	Single IV infusion	Small study; hypertension reported in prior metabolic cohorts	Direct target engagement but no ASCVD endpoint	NCT04975438
GSK1070806	Human anti-IL-18 IgG1 monoclonal antibody	Phase 2 terminated for futility; no safety concern identified	Atopic dermatitis	SC dose-ranging	Efficacy futility in a non-cardiovascular disease	Demonstrates target-specific clinical development risk	NCT05999799
Camoteskimab	Anti-IL-18 monoclonal antibody	Phase 2 completed	Atopic dermatitis	SC; dose-ranging	Results not yet fully published in peer-reviewed form	No cardiovascular efficacy evidence	NCT06436183
MAS825	Bispecific antibody neutralizing IL-1β and IL-18	Phase 2 active, not recruiting	Monogenic IL-18-driven autoinflammatory disease	Registry-defined dosing	Broader cytokine suppression may increase infection risk	Relevant to dual-cytokine strategy	NCT04641442
MAS825	Dual IL-1β/IL-18 blockade	Phase 2 completed; *n* = 31 combined trial	Coronary heart disease with CHIP	Single dose	Small biomarker study; no outcome efficacy conclusion	Most direct registered cardiovascular IL-18-pathway study	NCT06097663
DFV890	Upstream NLRP3 inflammasome inhibitor	Phase 2 completed; *n* = 31 combined trial	Coronary heart disease with CHIP	Oral daily	Suppresses IL-1β and IL-18; not target-specific	Tests upstream inflammasome modulation	NCT06097663
AZD4144	Oral inflammasome-pathway modulator; IL-6 primary PD and IL-18 secondary PD	Phase 1b completed; *n* = 29	ASCVD with CKD and hsCRP >2 mg/L	Oral for 28 days	Short exposure; no clinical outcome endpoint	Cardiovascular target-engagement study	NCT06675175

ASCVD, atherosclerotic cardiovascular disease; CHIP, clonal hematopoiesis of indeterminate potential; CKD, chronic kidney disease; IL-18BP, interleukin-18 binding protein; IV, intravenous; PD, pharmacodynamic; SC, subcutaneous.

A critical challenge is identifying patients in whom IL-18 is both active and causal. Unlike IL-1β, for which hsCRP enrichment was used in CANTOS, or IL-6, for which ziltivekimab trials enroll patients with residual inflammation, no validated biomarker stratifies cardiovascular patients for IL-18 inhibition (75). Ziltivekimab is a fully human monoclonal antibody that neutralizes the IL-6 ligand. Its development illustrates a more mature biomarker-to-outcome pathway than currently exists for IL-18.

Patient stratification is further complicated by IL-18 biochemistry. Total IL-18 is stable and readily measured, but most circulating IL-18 is bound to IL-18BP and biologically inactive (23, 76). Free IL-18 is more relevant but technically difficult to quantify and assay-dependent. For atherosclerosis, circulating free IL-18 has not been validated against plaque concentration, cellular source, imaging-defined vulnerability, or outcomes. Local endothelial, smooth-muscle-cell, and macrophage production may create compartmentalized activity that serum measurements miss. Standardized pre-analytical handling, calibrated free IL-18 assays, concurrent IL-18BP measurement, and tissue–plasma correlation are prerequisites for biomarker-guided trials.

Beyond biomarker challenges, preclinical models impose additional constraints on translational interpretation. Mouse models cannot fully replicate human atherosclerosis characteristics, which may limit the generalizability of preclinical findings to human populations. Interspecies differences in immune cell subsets, plaque composition, and disease duration risk overestimating the pathogenic role of IL-18 in human disease. To address these translational gaps, future studies should explore whether elevated serum free IL-18 identifies high-risk plaques, whether coronary sinus IL-18 levels—which reflect cardiac production—predict cardiovascular events, or whether IL-18/CXCL9 ratios, which improve diagnostic specificity for MAS, could be translated to cardiovascular risk stratification.

Before clinical implementation, chronic IL-18 inhibition requires dedicated safety evaluation. IL-18 contributes to defense against intracellular pathogens, NK-cell and CD8+ T-cell function, epithelial-barrier homeostasis, vaccine responses, and tumor immune surveillance. Short inflammatory-disease trials and genetic analyses cannot exclude uncommon infections, impaired vaccine responses, viral reactivation, or malignancy during years of cardiovascular treatment. Trials should therefore prespecify adjudicated serious infection, opportunistic infection, vaccine-response substudies, malignancy surveillance, and immune-cell pharmacodynamic monitoring. Safety may also differ between recombinant IL-18BP, monoclonal antibodies, dual cytokine blockade, and upstream inflammasome inhibition because of differences in half-life, tissue penetration, target stoichiometry, and breadth of pathway suppression.

Within the anti-inflammatory cardiovascular landscape, IL-18 remains less clinically validated than IL-1β, colchicine, or IL-6 inhibition. CANTOS demonstrated event reduction with canakinumab but increased fatal infection (2). COLCOT and LoDoCo2 showed lower ischemic events with low-dose colchicine after myocardial infarction and in chronic coronary disease, respectively, while also defining tolerability and infection signals at clinical scale (77, 78). Ziltivekimab markedly lowers inflammatory biomarkers and is being tested in phase 3 cardiovascular outcome programs; the ZEUS trial framework illustrates biomarker enrichment, hard endpoints, and long-term safety assessment (75, 84). By contrast, TNF inhibition has no established role in atherosclerotic event prevention, and CXCL9/CXCL10 and GM-CSF remain mechanistically relevant but clinically immature cardiovascular targets. IL-18 could eventually occupy a complementary niche, but head-to-head superiority should not be presumed.

## Emerging evidence and unresolved modifiers

Plaque heterogeneity limits any single-direction model of IL-18. Single-cell and spatial studies show that early, advanced, stable, and vulnerable lesions contain different endothelial, macrophage, smooth-muscle-cell, lymphocyte, and stromal states. Recent work has identified macrophage-like smooth muscle cells, proangiogenic endothelial populations, inflammatory cellular neighborhoods, and tertiary lymphoid structures within human plaques (81–83, 85). These atlases refine the cellular map but do not yet establish which cells produce biologically active free IL-18, which express functional receptor complexes, or whether IL-18-associated states are causal. Future studies should quantify IL18, IL18R1, IL18RAP, IL18BP, inflammasome components, and downstream IFN-γ/CXCL programs at single-cell and spatial levels with protein validation.

Sex and age are underexamined modifiers. Sex hormones, adiposity, immune-cell composition, and menopause can alter inflammatory signaling and plaque phenotype, yet most IL-18 studies are not powered for sex-by-IL-18 interactions. Aging introduces immunosenescence, inflammaging, clonal hematopoiesis, impaired efferocytosis, and altered T- and B-cell repertoires (79). These processes could change both benefit and harm from IL-18 blockade. Trials should stratify randomization by sex and age, report interaction estimates, and prospectively genotype common clonal hematopoiesis drivers rather than treating these variables as simple covariates.

Metabolic disorders may amplify or confound IL-18 associations. Obesity, insulin resistance, type 2 diabetes, metabolic syndrome, and metabolic dysfunction-associated steatotic liver disease increase inflammasome activity and cardiovascular risk; IL-18 is also implicated in beta-cell stress and diabetic complications (45, 46, 55, 80). Remnant-lipoprotein analyses further connect dyslipidemia with systemic inflammatory pathways, although they do not prove that IL-18 mediates plaque events (59). These conditions may identify an IL-18-high phenotype, but they also raise total IL-18 through pathways not specific to plaque activity. Mechanistic mediation, not correlation alone, is needed before metabolic enrichment is used for trial selection.

Adaptive immune checkpoints add another layer of uncertainty. IL-18 can promote cytotoxic lymphocyte activity and IFN-γ-dominant responses, while IL-18BP acts as a soluble checkpoint. Interactions with exhausted plaque T cells, regulatory T-cell stability, antigen presentation, and tertiary lymphoid structures remain incompletely resolved. Direct inhibition may reduce inflammatory effector activity but could also impair immune surveillance or alter efferocytosis. These competing consequences should be examined in human plaque explants and longitudinal multi-omic studies before combination immunomodulation is attempted.

## Conclusions and stage-gated future directions

IL-18 is biologically positioned at the intersection of inflammasome activation, vascular-cell stress, type 1 immunity, and metabolic inflammation. However, the evidence is not uniformly pro-atherogenic: plaque-tissue studies and many conventional mouse models support pathogenic activity, large circulating-biomarker cohorts show limited independent value, MR supports selected outcomes rather than coronary disease broadly, and JAK2V617F clonal hematopoiesis reveals potentially harmful consequences of neutralization. IL-18 should therefore be described as a plausible, context-dependent, and clinically unvalidated cardiovascular target.

A stage-gated development strategy is warranted. First, analytical studies should standardize free IL-18 and IL-18BP assays and establish how plasma measurements relate to tissue activity and imaging-defined plaque vulnerability. Second, prospective cohorts should prespecify sex, age, ancestry, metabolic disease, plaque phenotype, and clonal hematopoiesis, with external validation of biomarker thresholds. Third, early cardiovascular trials should demonstrate target engagement, dose–exposure relationships, tissue penetration, and directional effects on free IL-18, CXCL9/CXCL10, imaging markers, and mechanistically aligned endpoints before advancing to outcome trials. Fourth, randomized trials should use hard cardiovascular endpoints, active or add-on comparators where justified, and long-term infection, vaccine, and malignancy surveillance. Combination therapy with lipid lowering, colchicine, IL-6 inhibition, or other anti-inflammatory agents should be tested only after pharmacodynamic overlap and additive immunosuppression are characterized. These steps would distinguish biological plausibility from clinically useful precision anti-inflammatory therapy.
